# KIF26B and CREB3L1 Derived from Immunoscore Could Inhibit the Progression of Ovarian Cancer

**DOI:** 10.1155/2024/4817924

**Published:** 2024-02-13

**Authors:** Shanshan Cong, Yao Fu, Xibo Zhao, Qiuyan Guo, Tian Liang, Di Wu, Jing Wang, Guangmei Zhang

**Affiliations:** ^1^Department of Gynecology, Affiliated Women's Hospital of Jiangnan University, Wuxi, China; ^2^Department of Pharmacy, The Affiliated Wuxi People's Hospital of Nanjing Medical University, Wuxi, China; ^3^Department of Gynecology, The First Affiliated Hospital of Harbin Medical University, Harbin, China

## Abstract

**Background:**

Ovarian cancer (OV) is characteristic of high incidence rate and fatality rate in the malignant tumors of female reproductive system. Researches on pathogenesis and therapeutic targets for OV need to be continued. This study mainly analyzed the immune-related pathogenesis and discovered the key immunotherapy targets for OV.

**Methods:**

WGCNA was used for excavating hub gene modules and hub genes related to the immunity of OV. Enrichment analysis was aimed to analyze the related pathways of hub gene modules. Biological experiments were used for exploring the effect of hub genes on SKOV3 cells.

**Results:**

We identified two hub gene modules related to the immunoscore of OV and found that these genes in the modules were related to the extracellular matrix and viral infections. At the same time, we also discovered six hub genes related to the immunity of OV. Among them, KIF26B and CREB3L1 can affect the proliferation, migration, and invasion of SKOV3 cells by the Wnt/*β*-catenin pathway.

**Conclusions:**

The local infection or inflammation of ovarian may affect the immunity of OV. KIF26B and CREB3L1 are expected to be potential targets for the immunotherapy of OV.

## 1. Background

OV is the most lethal gynecological malignant tumor in women. According to the cancer statistics of 2023, the estimated new OV cases are 19,710 and the deaths are 13,270; in the meantime, OV has the highest fatality rate (67.33%) in all gynecological tumors [1]. The current standard treatment for OV is surgery combined with chemotherapy of platinum and paclitaxel. However, the OV cells have already spread to the pelvic or abdominal cavity when patients are initially diagnosed, which increases the difficulty of therapy and the recurrence rate and leads to a reduced survival rate [2]. Although new treatment methods such as targeted therapy have been applied to OV, the 5-year survival rate of OV has not been significantly improved. Therefore, we urgently need to conduct further research on OV to explore the pathogenesis from a new perspective and investigate more innovative and suitable therapeutic targets for OV.

Existing studies consider that immunity is crucial and complicated in the occurrence and development of tumors. At the same time, immunity also discloses a new direction for tumor therapy. For the moment, immunotherapy is mainly applied in melanoma and non-small-cell lung cancer, and the main targets of immunotherapy are checkpoint inhibitors (PD1, PDL1, and CTLA4), cytokines for lymphocyte promotion (recombinant IL-2 and recombinant INF*α*), and so on [3]. However, the research on immunotherapy for OV has not achieved significant progress. Therefore, we hope to find new immunotherapy targets to improve the prognosis of OV. During the research, we also must consider the role of the tumor microenvironment (TME) of OV because the TME will bring about a certain impact on the local immune status changing the intrinsic biologic characteristic of OV [4].

In our published study, we had evaluated the immune characterization and the role of TME cells in OV and established a model to calculate the immunoscore. Further evaluation of the immune risks had pointed out that patients with higher immunoscore have higher immune risk and poorer prognosis. Immunoscore gains the highest AUC in predicting the 5-year survival rate of patients, compared with the other clinicopathological characteristics of OV [5]. Therefore, we demonstrated that the immunoscore could be used as a potential prognostic biomarker of OV. In the present research, we conducted further analysis of the immunoscore aiming to explore the role of immunity, reveal the potential pathogenic mechanisms, and discover the potential immunotherapy targets of OV.

## 2. Materials and Methods

### 2.1. Data Downloading and Processing

The RNA sequencing data and clinicopathological features of OV in The Cancer Genome Atlas (TCGA) were downloaded from UCSC Xena (http://xena.ucsc.edu). The immunoscore of TCGA OV (*n* = 172) was acquired from our previous study [5]. The median absolute deviation values of genes were calculated and the top 5,000 genes were then screened for the next analysis. Finally, a total of 172 TCGA OV data with detailed clinicopathological features (OS, event, stage, grade, age, lymph node metastasis, and residual tumor diameter) and immunoscore were obtained. GSE18520 and GSE26712 were downloaded from Gene Expression Omnibus (GEO) (https://www.ncbi.nlm.nih.gov/geo), both of which included normal ovarian tissues and OV tissues, and the numbers of these two datasets are 63 and 195, respectively. RMA method in affy package [6] was used for normalizing the raw GEO data.

### 2.2. WGCNA

WGCNA package [7] was used for weighted gene coexpression network analysis (WGCNA) in our research. First, we deleted the OV samples with outliers in 172 TCGA samples. Then, the samples and the corresponding clinical phenotypes were clustered. To make the gene network in line with the nonscale network, we calculated the scale-free topology model fit *R*^2^ and mean connectivity to select the appropriate soft threshold *β*, setting standard for selecting *β* as follows: the *R*^2^ equals 0.9 and the mean connectivity is less than 100.

Thus, the weighted correlation analysis was performed on genes according to *β*. Then, we clustered the weighted gene matrix and merged the modules with high similarity for identifying gene modules. The correlation between gene modules and different clinical phenotypes of OV was calculated. Hub gene modules have the characteristic of the most significant correlations (including positive and negative correlations) with the immunoscore. In the end, the gene significance (GS) and the module membership (MM) were calculated, of which the selection criteria for the key genes in hub gene modules were GS > 0.2 and MM > 0.8 [8].

### 2.3. Enrichment Analysis

To study the functions of the genes in hub gene modules, gene ontology (GO) terms and Kyoto encyclopedia of genes and genomes (KEGG) pathways enrichment analyses were performed by ClusterProfiler package [9] in the research. The preponderant GO terms (including biological process, cellular component, and molecular function) and KEGG pathways in the analysis results were visualized.

### 2.4. PPI

Protein–protein interaction (PPI) analysis on these genes was performed in Cytoscape software to establish PPI network and to explore the interactions amongst. Valuable subnets with more closely related genes were selected from the whole PPI network by MCODE app [10] in Cytoscape. The strength of the relationship between these genes was visualized by the weight value calculated in WGCNA.

### 2.5. Identification of DEGs in OV

The differentially expressed genes (DEGs) between the normal ovarian group and OV group in GSE18520 and GSE26712 were, respectively, analyzed by limma package [11]. The DEGs with *P* < 0.05 in the results were selected. The intersections of the up-regulated DEGs and down-regulated DEGs in these two datasets were used for filtrating the hub genes related to the immunity of OV.

### 2.6. Cell Culture and Treatment

SKOV3 cell line was purchased from the Procell (Wuhan, China). The cells were cultured in 25 cm^2^ flasks with Mccoy's 5a Medium from Yuanpei (#L630KJ, Shanghai, China) supplemented with 10% fetal bovine serum (#S711-001S, Lonsera), 100 U/ml of penicillin G, and 100 U/ml of streptomycin at 37°C in 5% CO_2_ and 95% air. Before treatments, the cells were seeded into appropriate plates and maintained serum-free for 12 hr.

### 2.7. RNA Interference

The specific small interfering RNA (siRNA) for KIF26B, CREB3L1, and a negative control siRNA were acquired from GenePharm (Shanghai, China). SKOV3 cells (2 × 10^5^ per well) were cultured in a six-well plate and transfected with final concentration of 100 nM siRNA using lipofectamine 2000 (Invitrogen, CA, USA), according to the manufacturer's instructions. Forty-eight hours after transfection, the cells were harvested for further experiments. The sequences of siRNAs are shown in Table 1.

### 2.8. Quantitative RT-PCR

Total RNA was extracted from SKOV3 cells using Trizol (Invitrogen, Carlsbad, CA). As described in our previous work [12], qRT-PCR was carried out on a Roche LightCycler® 480 II Real-Time PCR System using SYBR Green realtime PCR Master Mix (TOYOBO, TYB-QPK-201). After the reactions were complete, the comparative threshold cycle (Ct) method was used to calculate the relative gene (KIF26B and CREB3L1) expression. GAPDH was used as an internal control. qPCR primers were purchased from Sangon Biotech (Shanghai, China). The primers sequences are shown in Table 2.

### 2.9. Cell Viability Assay

#### 2.9.1. MTT Assay

The cells were seeded in 96-well plates with a density of 5 × 10^4^. The transfection was carried out after the cells adhered to the wall and the fresh medium with 10% FBS was replaced after 4 hr. After 48 hr, 3-(4,5-dimethylthiazol-2-yl)-2,5-diphenyltetrazolium bromide (MTT) solution (5 mg/ml) was added to the well and continued to incubate for 4 hr. Then, the medium was poured out gently and 150 *μ*l of DMSO was added to fully dissolve the crystal. The optical density (OD) was read at 490 nm using a SpectraMax® Absorbance reader (Molecular Devices, San Jose).

#### 2.9.2. CCK8 Assay

The cell counting kit-8 (CCK8) was purchased from DOJINDO and used as the manual. Briefly, the cells were inoculated in 96-well plates at a density of 5 × 10^4^. Twenty-four hours later, the cells were transfected after being plastered and then replaced with 100 *μ*l of culture medium after 4–6 hr. After 48 hr, 10 *μ*l of CCK8 solution was added to each well and incubated in a CO_2_ incubator for 1 hr. Cell viability was calculated by measuring the absorbance value at 450 nm with a SpectraMax® Absorbance reader (Molecular Devices, San Jose).

### 2.10. Wound Healing Assay

The cells were seeded in six-well plates until the cells were full without gap. Using 200 *μ*l sterile plastic yellow tip to create a wound line across the surface of plates, the suspension cells were removed with PBS (0 hr). Cells were cultured in different conditions using different treatments at 37°C for 48 hr, and then images were taken with a phase-contrast microscope at the time point of 0, 24, and 48 hr. The wound healing rate was analyzed and calculated by Image J.

### 2.11. Cell Invasion Assay

SKOV3 cells were seeded into the upper layer of a Transwell membrane insert with an 8 *μ*m pore size in a 24-well plate (Corning). The membranes were coated with Matrigel (BD Biosciences, Franklin Lakes, NJ, USA) for invasion assays. Then, the medium containing 10% FBS was put in the bottom chamber as an attractant. After 24 hr of invasion, the cells were fixed with methanol and stained with 0.2% crystal violet. The cell numbers were counted using FIJI (Image J2).

### 2.12. Western Blot Analysis

Total protein was extracted by Ripa lysate (P0013B, Beyotime, China) according to the manufacturers' instructions. The isolated protein (40 *μ*g) was separated by 10% SDS-PAGE and transferred onto an NC membrane (Bio-Rad Laboratories, Hercules, CA, USA). The primary antibodies were incubated on the membranes overnight at 4°C. The primary antibodies used in the present study included anti-PCNA antibody (Proteintech, #10205-2-AP, 1 : 1,000 dilution), anti-Wnt5 a/b antibody (Proteintech, #55184-1-AP, 1 : 1,000 dilution), anti-*β* catenin antibody (Proteintech, #51067-2-AP, 1 : 1,000 dilution), and anti-GAPDH antibody (Proteintech, #10494-1-AP, 1 : 2,000 dilution). Subsequently, the membranes were washed with TBS-0.1% Tween 20 (TBST) and incubated with goat anti-rabbit (IgG) IRDye® 800 CW (LI-COR, P/N 926-32211, 1 : 10,000 dilution) at room temperature for 1 hr. The bands were quantified using the Odyssey infrared imaging system (LI-COR) and Odyssey v3.0 software (LI-COR, Lincoln, Nebraska, USA).

### 2.13. Statistical Analysis

Bioinformatics analyses in the research were performed in R Studio 3.6 (with R 3.6.0 and Java 10.0.2). Pearson method was used for calculating the correlation coefficient. In the experimental part, FIJI (Image J2) was used for image analysis, and Graphpad Prism 9 was used for drawing statistics. The statistical method was one-way ANOVA followed by the Dunnett test for multiple comparisons. *P* < 0.05 was considered as statistically significant in the entire analysis.

## 3. Results

### 3.1. Identification of Two Hub Gene Modules Related to Immunoscore by WGCNA in OV

In previous studies, we found that the immunoscore had a key role in predicting the prognosis of OV. Therefore, we would detect hub gene modules that are closely related to immunoscore by performing WGCNA on 172 TCGA OV data with 5,000 genes (Supplementary 2). An outlier sample was found and deleted when clustering OV samples for the first time. Subsequently, we performed a second clustering of 171 OV samples and integrated the corresponding clinical phenotypes (OS, event, stage, grade, age, lymph node metastasis, residual tumor diameter, and immunoscore) (Supplementary 1).

**Figure 1:**
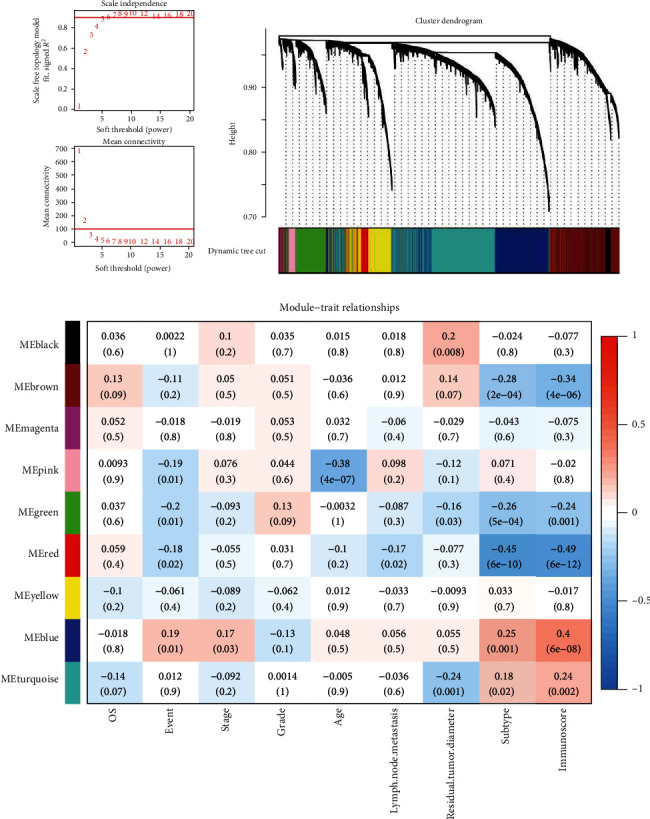
Identification of gene modules related to the immunoscore of OV. (a) The filtering of soft threshold by *R*^2^ and mean connectivity. (b) The cluster dendrogram divided genes into nine modules. (c) The correlation between gene modules and the clinical phenotype of OV.

According to the *R*^2^ and mean connectivity, we selected *β* = 6 (*R*^2^ = 0.9) as the soft threshold to establish a network closer to the scale-free network (Figure 1(a)). The weighted correlation analysis was performed on 5,000 genes and dynamic hybrid cutting could gather genes with similar expression models. As a result, the genes were divided into nine gene modules with different gene numbers: black (*n* = 102), blue (*n* = 1,071), brown (*n* = 947), green (*n* = 433), magenta (*n* = 37), pink (*n* = 101), red (*n* = 175), turquoise (*n* = 1,522), and yellow (*n* = 612) (Figure 1(b)).

The correlation coefficients and corresponding *P*-value between the eight clinical phenotypes and nine gene modules were calculated. In all clinical phenotypes of OV, we found that the immunoscore had the highest positive correlation coefficient (0.4, *P*=6e − 08) with the blue module and the highest negative correlation coefficient (−0.49, *P*=6e − 12) with the red module (Figure 1(c)). Therefore, we considered the blue and the red modules as hub gene modules related to the immunoscore of OV.

### 3.2. Extracellular Matrix and Viral Infections Were Relevant to the Genes in Hub Gene Modules

**Figure 2:**
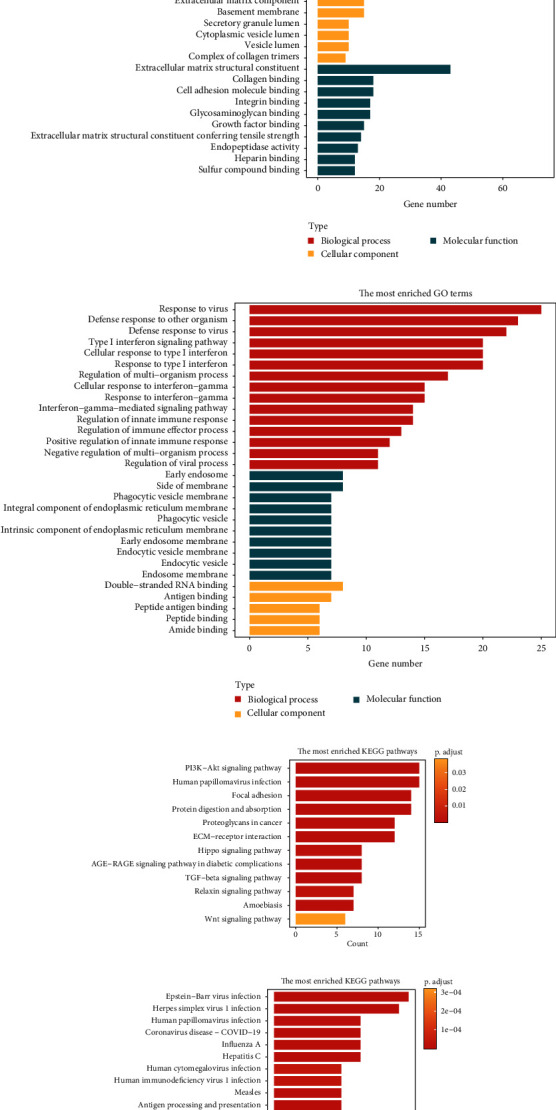
The enrichment analysis of hub gene modules. (a) The GO function annotation enrichment analysis of the blue module. (b) The GO function annotation enrichment analysis of the red module. (c) The KEGG pathway enrichment analysis of the blue module. (d) The KEGG pathway enrichment analysis of the red module.

GO terms and KEGG pathways enrichment analyses were carried out on genes in hub gene modules. We first performed the GO terms enrichment analysis. The results showed that the genes in the blue module were mainly enriched in the extracellular matrix organization, extracellular structure organization, extracellular matrix, collagen-containing extracellular matrix, extracellular matrix structural constituent, and other extracellular matrix-related items (Figure 2(a)). The enriched GO terms of the red module were significantly related to inflammatory reactions, such as response to virus, defense response to other organisms, defense response to virus, type I interferon signaling pathway, cellular response to type I interferon, and response to type I interferon (Figure 2(b)).

Subsequently, the KEGG pathways enrichment analysis was performed on these two modules. In the blue module, the main enriched KEGG pathways were the PI3K-Akt signaling pathway and human papillomavirus (HPV) infection (Figure 2(c)). Some virus-related KEGG pathways were also enriched in the red module, such as Epstein–Barr (EB) virus infection and herpes simplex virus 1 (HSV1) infection (Figure 2(d)).

**Figure 3:**
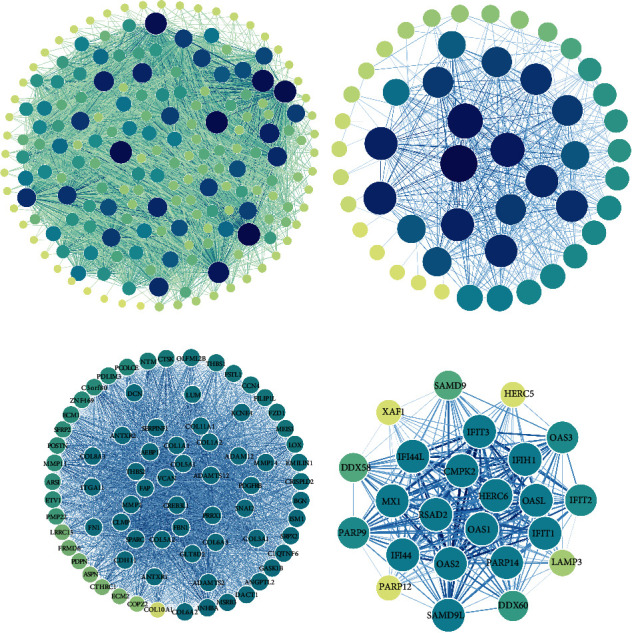
The PPI networks of hub gene modules. (a) The PPI overall network of the blue module. (b) The PPI overall network of the red module. (c) The PPI subnetwork of the blue module. (d) The PPI subnetwork of the red module.

We constructed and visualized PPI networks of the blue module and the red module in Cytoscape software based on the degrees and weights of genes calculated in WGCNA (Figures 3(a) and 3(b)). Inextricable connections were exhibited in these two networks. Then, two subnets were, respectively, extracted from the two whole PPI networks. The subnet of the blue module concluded 75 nodes and 2,664 edges, and many genes in the subnet (such as CREB3L1, COL5A1, MMP14, PRRX1, and ADAM12) were related to each other or tumors [13–17] (Figure 3(c)). The subnet of the red module concluded 24 nodes and 259 edges, and many members in the 2′-5′-oligoadenylate synthetases (OAS) family (such as OAS1, OAS2, OAS3, and OASL) appeared in the subnet (Figure 3(d)).

### 3.3. Identification of Six Hub Genes Related to the Immunity of OV

**Figure 4:**
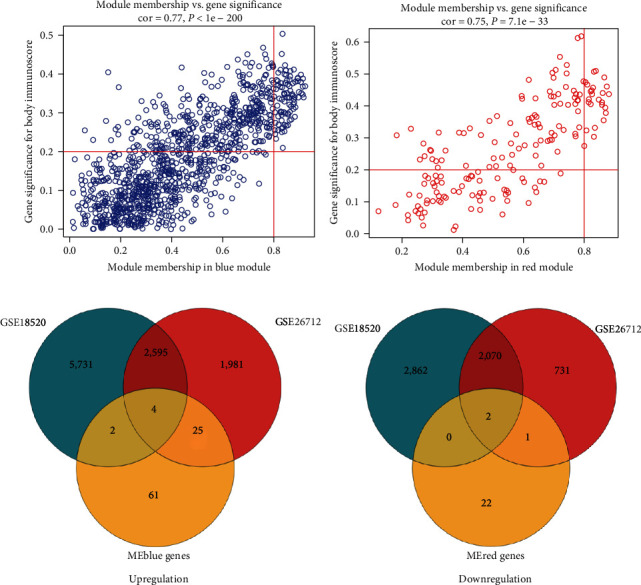
Identification of hub genes related to the immunity of OV. (a) Identification of key genes in the blue module. (b) Identification of key genes in the red module. (c) The intersection of up-regulated DEGs in GSE18520 and GSE26712 with key genes in the blue module. (d) The intersection of down-regulated DEGs in GSE18520 and GSE26712 with key genes in the red module.

We filtrated the key genes in hub gene modules by the GS and MM of genes in the blue and the red modules. As a result, 92 key genes in the blue module and 25 key genes in the red module were obtained (Figures 4(a) and 4(b), Supplementary 3). Then, we performed the differential analysis of the gene expression in the GSE18520 and GSE26712 datasets by limma package. In GSE18520, we got 13,266 DEGs (8,332 up-regulated DEGs and 4,934 down-regulated DEGs in OV). In GSE26712, we got 7,409 DEGs (4,605 up-regulated DEGs and 2,804 down-regulated DEGs in OV) (Supplementary 4).

Because the blue module was positively correlated with immunoscore and the red module was negatively correlated with immunoscore significantly, we intersected the key genes in the blue module with up-regulated DEGs in GSE18520 and GSE26712 and intersected the key genes in the red module with down-regulated DEGs in GSE18520 and GSE26712. Finally, we got four up-regulated hub genes (LOXL2, LZTS1, KIF26B, and CREB3L1) in the blue module (Figure 4(c)) and two down-regulated hub genes (TRIM22 and DDX60) in the red module (Figure 4(d)). These genes were not only DEGs in OV tissues and normal ovarian tissues but also key genes in hub gene modules related to the immunoscore of OV, so we regarded them as the hub genes related to the immunity of OV.

### 3.4. Knockdown of KIF26B and CREB3L1 Gene Expression Inhibited the Proliferation, Migration, and Invasion of SKOV3 Cells

**Figure 5:**
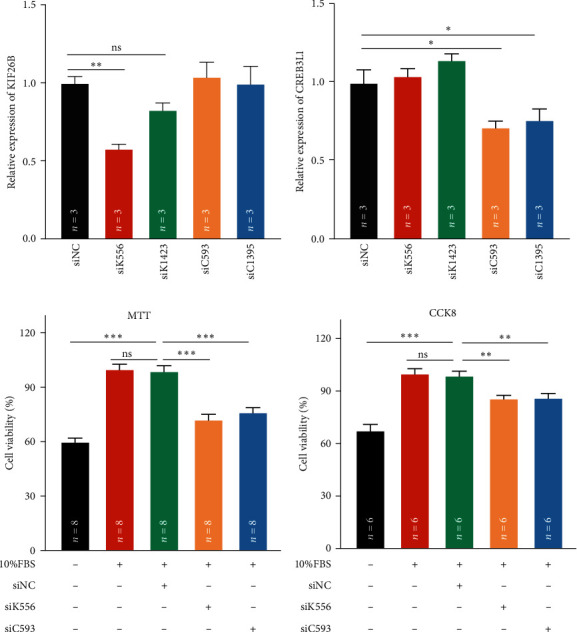
Knockdown of KIF26B and CREB3L1 gene expression inhibited the proliferation of SKOV3 cells. (a) The detection of the expression of KIF26B by qRT-PCR when SKOV3 cells were transfected with 24 hr. (b) The detection of the expression of CREB3L1 by qRT-PCR when SKOV3 cells were transfected with 24 hr. (c) The detection of the proliferation activity of SKOV3 cells by MTT when the expression of KIF26B and CREB3L1 was decreased. (d) The detection of the proliferation activity of SKOV3 cells by CCK8 when the expression of KIF26B and CREB3L1 was decreased. The *n* in the figure represented the number of repeated experiments. The statistical method was one-way ANOVA followed by Dunnett test for multiple comparisons. (ns: nonsignificant,  ^*∗*^*P* < 0.05,  ^*∗∗*^*P* < 0.01,  ^*∗∗∗*^*P* < 0.001).

The biological behavior of SKOV3 cells was analyzed after knocking down the expression of KIF26B and CREB3L1. We first screened the appropriate siRNA (small interference RNA) sequence for KIF26B and CREB3L1 by qRT-PCR. The results showed that siK556 and siC593 could significantly knock down the gene expression of KIF26B and CREB3L1 respectively, and they were used in the subsequent experiments of SKOV3 cells (Figures 5(a) and 5(b)). The cell viability assays (MTT and CCK8) revealed that knockdown of KIF26B and CREB3L1 could decrease the cell viability of SKOV3 cells (Figures 5(c) and 5(d)). Therefore, we considered that knockdown of KIF26B and CREB3L1 could inhibit the proliferation of SKOV3 cells.

**Figure 6:**
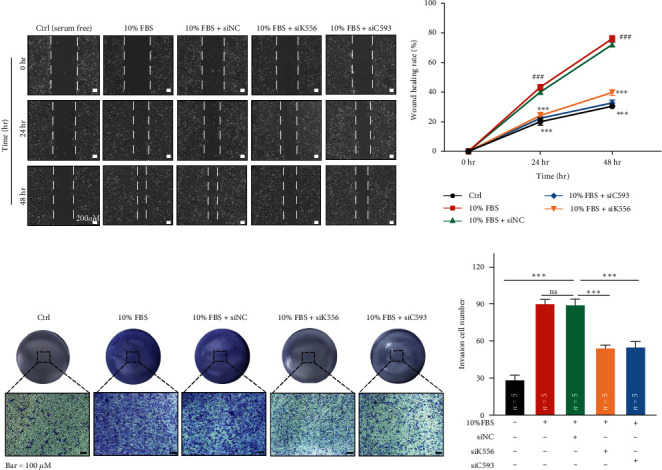
Knockdown of KIF26B and CREB3L1 gene expression inhibited the migration and invasion ability of SKOV3 cells. (a) The detection of the wound area after 0, 24, and 48 hr of wound and transfection by wound healing. (b) The statistics of wound healing rate after 0, 24, and 48 hr of wound and transfection. The scale bar was 200 *μ*m, the number of repeated experiments was six, and the statistical method was one-way ANOVA followed by Dunnett test for multiple comparisons. (###*P* < 0.001, Ctrl vs. 10% FBS + siNC group.  ^*∗∗∗*^*P* < 0.001,10% FBS + siNC vs. 10% FBS + siK556 or 10% FBS + siC593 group.) (c) The detection of the crystal violet staining of SKOV3 cells that enter the lower chamber by the transwell invasion assays when the expression of KIF26B and CREB3L1 was decreased. (d) The statistical quantification of the results of transwell invasion assay. The scale bar was 100 *μ*m, and the *n* in the figure represented the number of repeated experiments. The statistical method was one-way ANOVA followed by Dunnett test for multiple comparisons. (ns: nonsignificant,  ^*∗∗∗*^*P* < 0.001).

Then, we continued to detect if KIF26B and CREB3L1 could affect the migration and invasion of SKOV3 cells by wound healing assay and cell invasion assay. The results of the wound healing assay showed that knockdown of KIF26B and CREB3L1 increased the wound healing rate of SKOV3 cells after culturing 24 and 48 hr, which meant that knockdown of KIF26B and CREB3L1 could inhibit the migration of SKOV3 cells (Figures 6(a) and 6(b)). The results of cell invasion assay exhibited that knockdown of KIF26B and CREB3L1 reduced the invasion cell number of SKOV3 cells significantly (Figures 6(c) and 6(d)). Therefore, we considered that knockdown of KIF26B and CREB3L1 could inhibit the invasion of SKOV3 cells.

### 3.5. KIF26B and CREB3L1 Influenced the Proliferation, Migration, and Invasion of SKOV3 Cells through PCNA and Wnt/*β*-Catenin Pathway

**Figure 7:**
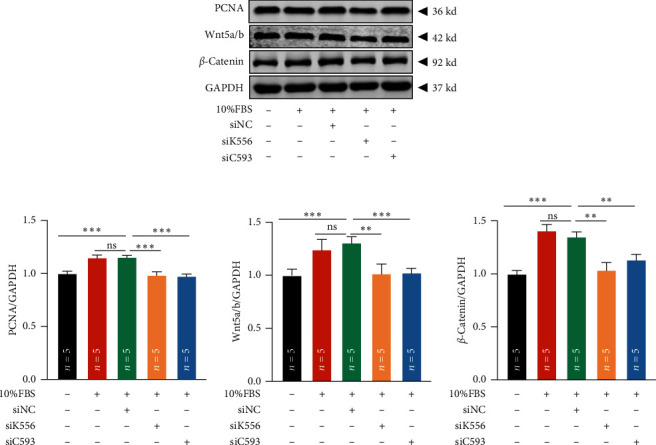
Knockdown of KIF26B and CREB3L1 gene expression inhibited the expression of PCNA and the Wnt/*β*-catenin signaling pathway. (a) The detection of the expression levels of PCNA, Wnt5 a/b, and *β*-catenin protein by WB when the expression of KIF26B and CREB3L1 was decreased. (b) The statistical quantification of the results of WB. The *n* in the figure represented the number of repeated experiments. The statistical method was one-way ANOVA followed by Dunnett's test for multiple comparisons. (ns: nonsignificant,  ^*∗∗*^*P* < 0.01,  ^*∗∗∗*^*P* < 0.001).

We had known that KIF26B and CREB3L1 were related to the proliferation, migration, and invasion of SKOV3 cells. We therewith conducted further research on the related mechanisms. Proliferating cell nuclear antigen (PCNA) is a protein with a molecular weight of 36 KD. It is involved in the initiation of cell proliferation and is an indicator of cell proliferation status [18, 19]. At the same time, researchers had reported that the Wnt/*β*-catenin signaling pathway was involved in the proliferation, migration, and invasion of a variety of tumors [20–23]. Therefore, the expression of PCNA, Wnt5 a/b, and *β*-catenin protein was detected by WB (western blot) assay in SKOV3 cells with knockdown of KIF26B and CREB3L1 gene expression. The results showed that knockdown of KIF26B and CREB3L1 could reduce the expression of PCNA, Wnt5 a/b, and *β*-catenin (Figures 7(a) and 7(b)). On the whole, we speculated that knockdown of KIF26B and CREB3L1 could inhibit the proliferation, migration, and invasion of OV through regulating PCNA and Wnt/*β*-catenin pathway.

## 4. Discussion

Although 379 TCGA OV data were downloaded from UCSC Xena, our previous studies believed that the immunoscore had a more significant role in predicting the prognosis of OV in 172 TCGA samples. To find hub gene modules related to the immunoscore of OV more accurately, we chose the same 172 TCGA samples in this present research. In the WGCNA, we got a total of nine gene modules and found that the immunoscore had the highest correlation coefficient among all phenotypes (stage, grade, age, lymph node metastasis, and residual tumor diameter), which proved the advantage of immunoscore. At the same time, we also discovered two hub gene modules with the highest absolute value of the correlation coefficient in the immunoscore.

In the enrichment analysis of the blue module, we found that the enriched GO terms were mainly extracellular matrix-related items. The extracellular matrix can contribute to tumorigenesis and tumor metastasis by promoting the occurrence of EMT [24], glycometabolism [25], or other pathways. Studies of cancer therapeutics have begun to focus on the extracellular matrix [26]. At the same time, the main enriched KEGG pathways were the PI3K-Akt signaling pathway and human papillomavirus (HPV) infection. The PI3K-Akt signaling pathway is a classic cancer-promoting pathway, which plays an active role in the progression of multiple cancers including OV and many inhibitors of this pathway have been used in the clinical trials of the therapy for OV [27]. HPV infection is the main cause of cervical cancer. However, studies had shown that HPV DNA was found in 74% of OV tissues, and its expression levels were significantly higher than that in benign ovarian tissues [28]. Therefore, we have reasons to believe that HPV infection may also be a potential factor in the occurrence of OV.

In the enrichment analysis of the red module, the enriched GO terms and KEGG pathways were mainly a variety of viral infections-related inflammation items. Virus infections could induce the production of various immune cells with the function of antitumor to clear the tumor cells. Surprisingly, the COVID-19 pathways appeared in the enriched results. One of the possible reasons is that this new virus may indeed be found to be related to OV in the future. Another reason is that the infection of this virus could produce a response similar to the infection of other viruses. Nevertheless, we can still speculate that local infection or inflammation of the ovarian may affect the immunity of OV.

The PPI analysis exhibited that the genes in hub modules were closely related. In the blue module, a gene had an average of 35 genes related to it, which helped in the discovery of valuable gene pairs. In the subnet of the red module, we found multiple members of the OAS family. The OAS family is an antiviral enzyme induced by interferon [29]. Studies showed that OAS1 could survive through DNA damage preventing the death of tumor cells [30]. In trastuzumab-resistant gastric cancer, OAS1, OAS2, OAS3, and OASL were all identified as key genes [31]. Therefore, the specific mechanism of the OAS family in OV is worthy of further exploration.

Six hub genes related to the immunity of OV were acquired in the research by intersecting key genes in hub gene modules and DEGs of OV, which could ensure that these hub genes had the largest capabilities. LOXL2 is a kind of secretases that catalyzes collagen cross linking and it plays a vital role in developmental angiogenesis. Studies had shown that LOXL2 was elevated in the plasma of patients with OV [32], and the inhibitors of LOXL2 could enhance the antitumor effect of chemotherapy in OV [33]. LOXL2 is also a potential biomarker of poor prognosis for OV simultaneously [34]. LZTS1 gene is located on chromosome 8p22. Califano et al. [35] considered that LZTS1 had a certain relationship with the FIGO stage and LZTS1 also could predict the therapeutic response of OV patients under paclitaxel chemotherapy.

KIF26B is a member of the human kinesin family. The up-regulation of KIF26B is involved in the occurrence of tumors and is associated with the tumor diameter, metastasis, and poor prognosis in breast cancer [36], gastric cancer [37], and colorectal cancer [38]. CREB3L1 is a cAMP response element binding protein, which could predict the response of triple-negative breast cancer with doxorubicin chemotherapy [39]. The overexpression of CREB3L1 also could be used as a diagnostic biomarker of the myeloproliferative tumor with negative Philadelphia chromosome [40]. However, the functions of CREB3L1 in OV have not been clarified yet.

As a transcriptional regulator involved in various biological processes, TRIM22 plays the role of E3 ubiquitin ligase [41]. The expression of TRIM22 is reduced in tumor tissues, and the overexpression of TRIM22 could inhibit the migration, invasion, proliferation, and cell cycle activity in endometrial cancer [42]. DDX60 is a member of RNA lyase and also a transcription factor. The transcription product of DDX60 gene plays a crucial role in the antiviral activity and interferon immunity [43]. At the same time, DDX60, as a new type of antiviral helicase, is the outpost of the cytoplasmic antiviral reaction, and it can participate in the degradation pathway of viral ribonucleic acid [44]. In addition, the low expression of DDX60 gene may also be related to the radio sensitivity of patients with breast cancer [45].

We randomly selected two genes (KIF26B and CREB3L1) for experimental verification. The results showed that knockdown of KIF26B and CREB3L1 expression could inhibit the proliferation, migration, and invasion of SKOV3 cells, which meant that KIF26B and CREB3L1 were related to the progression of OV. Although we have found that KIF26B and CREB3L1 could affect the proliferation, migration, and invasion of OV involving the Wnt/*β*-catenin signaling pathway, further researches are still needed to find out other potential and specific immune-related mechanisms. Even so, we still believe that the study of KIF26B and CREB3L1 lay the foundation for the immunotherapy of OV. At the same time, there still needs further experiments to verify the roles of other hub genes in the occurrence and development of OV.

## 5. Conclusion

In summary, we obtained two hub gene modules relevant to the immunoscore of OV and found that the genes in the hub gene module were mainly enriched on items related to the extracellular matrix and viral infection, which may affect the immunity of OV. Furthermore, we identified six hub genes related to the immunity of OV. Finally, biological experiments found that KIF26B and CREB3L1 participated in the progression of OV. Therefore, KIF26B and CREB3L1 are expected to be potential targets for the therapy of OV.

## Figures and Tables

**Table 1:** The RNA oligo sequences.

Gene	Species	RNA oligo (5′-3′)
KIF26B-556	Homo	Sense: GCUGGUACCGGAAAGCAUATT
		Antisense: UAUGCUUUCCGGUACCAGCTT

KIF26B-1423	Homo	Sense: CCCUGUACCCAUACCAGAUTT
		Antisense: AUCUGGUAUGGGUACAGGGTT

CREB3L1-593	Homo	Sense: GACCACUUUACGGAGAACATT
		Antisense: UGUUCUCCGUAAAGUGGUCTT

CREB3L1-1395	Homo	Sense: GUCGUAAGAAGGAGUATT
		Antisense: UACUCCUUCUUCUUACGACTT

Negative control	Homo	Sense: UUCUCCGAACGUGUCACGUTT
		Antisense: ACGUGACACGUUCGGAGAATT

**Table 2:** The primer sequences.

Gene	Species	Primer (5′−3′)
KIF26B	Human	Forward primer: TTCTCGGCTGTGATTCACGAC
		Reverse primer: AGGTGAGTGGCGCAAATGT

CREB3L1	Human	Forward primer: GCACCTGGACCACTTTACGG
		Reverse primer: AGCACAGGGTCATCAAAGAAG

GAPDH	Human	Forward primer: ACAACTTTGGTATCGTGGAAGG
		Reverse primer: GCCATCACGCCACAGTTTC

## Data Availability

The datasets used in the research were downloaded from http://xena.ucsc.edu and https://www.ncbi.nlm.nih.gov/geo.

## Abbreviations

DEGs: Differentially expressed genes
GEO: Gene Expression Omnibus
GO: Gene ontology
GS: Gene significance
HPV: Human papillomavirus
HSV1: Herpes simplex virus 1
KEGG: Kyoto Encyclopedia of Genes and Genomes
MM: Module membership
OD: Optical density
OV: Ovarian cancer
PCNA: Proliferating cell nuclear antigen
PPI: Protein–protein interaction
TME: Tumor microenvironment
TCGA: The Cancer Genome Atlas
WGCNA: Weighted gene coexpression network analysis.

## Supplementary Materials

Supplementary 1 The clustering of OV samples with the corresponding clinical phenotypes.

Supplementary 2 The 5,000 genes expression data and clinical phenotypes of 172 OV samples.

Supplementary 3 The key genes in the blue module and red module.

Supplementary 4 The DEGs of GSE18520 and GSE26712.
