# Supplementary material for: KIF26B and CREB3L1 Derived from Immunoscore Could Inhibit the Progression of Ovarian Cancer
Source: J Immunol Res. 2024 Feb 13;2024:4817924. doi: 10.1155/2024/4817924 (PMC10878761; doi:10.1155/2024/4817924)
Supplement: Supplementary 1 — The clustering of OV samples with the corresponding clinical phenotypes. [file 4817924.f1.pdf]

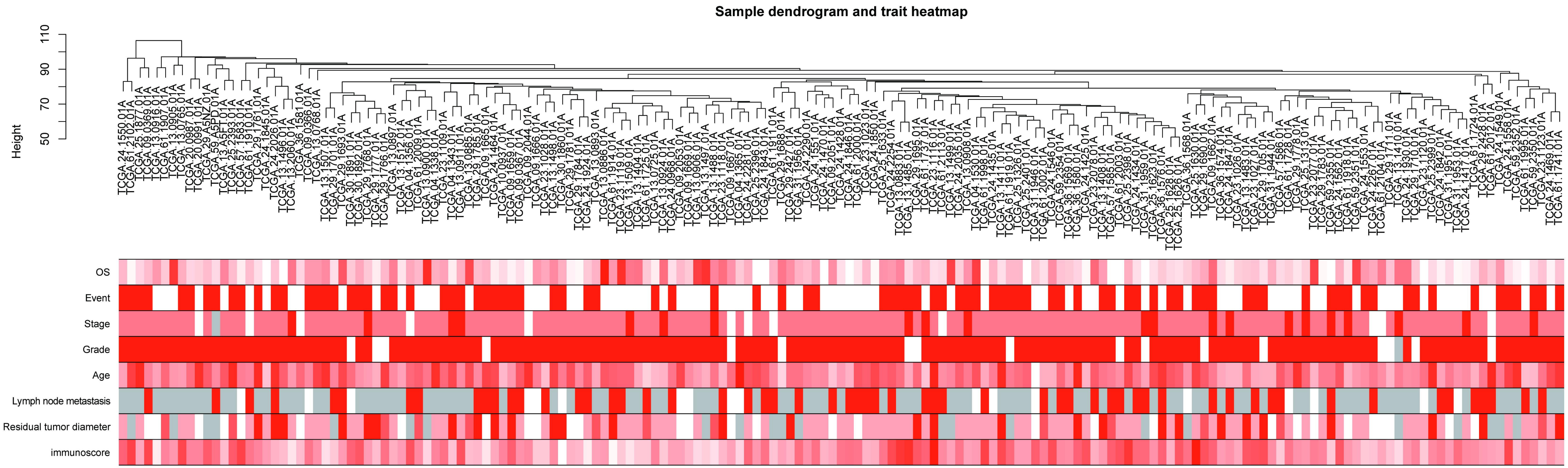

Figure S1. The clustering of OV samples with the corresponding clinical phenotypes.
